# VAREANT: a bioinformatics application for gene variant reduction and annotation

**DOI:** 10.1093/bioadv/vbae210

**Published:** 2024-12-31

**Authors:** Rishabh Narayanan, William DeGroat, Elizabeth Peker, Saman Zeeshan, Zeeshan Ahmed

**Affiliations:** Rutgers Institute for Health, Health Care Policy and Aging Research, Rutgers, The State University of New Jersey, New Brunswick, NJ 08901, United States; Rutgers Institute for Health, Health Care Policy and Aging Research, Rutgers, The State University of New Jersey, New Brunswick, NJ 08901, United States; Rutgers Institute for Health, Health Care Policy and Aging Research, Rutgers, The State University of New Jersey, New Brunswick, NJ 08901, United States; Department of Biomedical and Health Informatics, UMKC School of Medicine, Kansas City, MO 64108, United States; Rutgers Institute for Health, Health Care Policy and Aging Research, Rutgers, The State University of New Jersey, New Brunswick, NJ 08901, United States; Department of Medicine, Division of Cardiovascular Diseases and Hypertension, Robert Wood Johnson Medical School, New Brunswick, NJ 08901, United States

## Abstract

**Motivation:**

The analysis of high-quality genomic variant data may offer a more complete understanding of the human genome, enabling researchers to identify novel biomarkers, stratify patients based on disease risk factors, and decipher underlying biological pathways. Although the availability of genomic data has sharply increased in recent years, the accessibility of bioinformatic tools to aid in its preparation is still lacking. Limitations with processing genomic data primarily include its large volume, associated computational and storage costs, and difficulty in identifying targeted and relevant information.

**Results:**

We present VAREANT, an accessible and configurable bioinformatic application to support the preparation of variant data into a usable analysis-ready format. VAREANT is comprised of three standalone modules: (i) Pre-processing, (ii) Variant Annotation, (iii) AI/ML Data Preparation. Pre-processing supports the fine-grained filtering of complex variant datasets to eliminate extraneous data. Variant Annotation allows for the addition of variant metadata from the latest public annotation databases for subsequent analysis and interpretation. AI/ML Data Preparation supports the user in creating AI/ML-ready datasets suitable for immediate analysis with minimal pre-processing required. We have successfully tested and validated our tool on numerous variable-sized datasets and implemented VAREANT in two case studies involving patients with cardiovascular diseases.

**Availability and implementation:**

The open-source code of VAREANT is available at GitHub: https://github.com/drzeeshanahmed/Gene_VAREANT

## 1 Introduction

Advancements in genome sequencing technologies have resulted in an immense wealth of available genomic data. The analysis of genetic variations via genome-wide association studies (GWAS) can improve our understanding of disease prognosis, treatments, and etiology by helping to uncover disease-causing variants and complex gene-disease relationships (Uffelmann *et al.* 2021). Recent progress in artificial intelligence (AI) and machine learning (ML) techniques have demonstrated their efficacy for genomic predictive analysis (Ahmed *et al.* 2020). The introduction of AI/ML prediction tools in the field of genomics has increased our understanding of disease etiology and shows potential for further uptake in clinical practice (Lin *et al.* 2023). However, the rapid growth of genomic data presents many analytic obstacles. Most genomic data formats are not immediately suitable for AI/ML analyses, requiring extensive preprocessing. Although state-of-the-art bioinformatic tools have been created to individually support the various data transformation stages, there is a lack of tools to extract highly targeted genomic data into a format suitable for immediate analysis. In addition, large volumes of heterogenous genetic information make data preparation difficult for targeted studies investigating only specific genes of interest. These datasets often include thousands of irrelevant data points resulting in wasteful processing and lengthy computation times (Schadt *et al.* 2010). Moreover, such workflows are often inaccessible to clinicians and translational researchers that lack the necessary computational expertise (Stephens *et al.* 2015, Alvarez *et al.* 2021). Ultimately, addressing these challenges will help render genomic analyses more accessible, affordable, and effective.

Processing variant data is an arduous process with many stages: raw sequence files undergo numerous transformations such as quality checking, trimming, alignment to a reference genome, variant calling, filtering, annotation, analysis, and visualization. Many tools exist to streamline each of these stages, including Burrows-Wheeler Aligner (BWA) (Yang *et al.* 2009) for alignment, Genome Analysis Toolkit (GATK) (McKenna *et al.* 2010) for variant calling, and SnpEff (Cingolani 2022) or Ensembl Variant Effect Predictor (VEP) (McLaren *et al.* 2016) for annotation. To support the variant calling workflow, we have recently developed a reliable Java-based Whole Genome/Exome (JWES) Data Processing Pipeline (Ahmed *et al.* 2021). JWES centralizes these various tools into a single cohesive pipeline for processing variant files. Using BWA for alignment, GATK for variant calling, and SnpEff for annotation, JWES allows the user to easily prepare a Variant Call Format (VCF) dataset. The VCF format is an extensible data format to encode mutation data alongside corresponding annotations (Danecek *et al.* 2011), and it is widely supported by many genomic tools. To help address these data processing challenges, we have developed VAriant REduction and ANnoTation (VAREANT), a configurable and accessible bioinformatic tool to support the curation of targeted variant in AI/ML-ready datasets. VAREANT is designed as a series of standalone modules to support the user with their various data preparation needs. Notably, VAREANT provides a unifying interface for processing large volumes of genomic data and offers a more accessible method of preparing variant data for subsequent investigative analysis. It was validated in a case study with a selective cohort of patients with cardiovascular diseases (CVDs) to extract relevant CVD-associated variants and annotations. With its utilization in data management pipelines, VAREANT can help reduce barriers to handling genomic data by simplifying the data transformation workflow and can aid in producing effective datasets equipped for subsequent AI/ML analyses.

## 2 Methods

**Figure 1.**
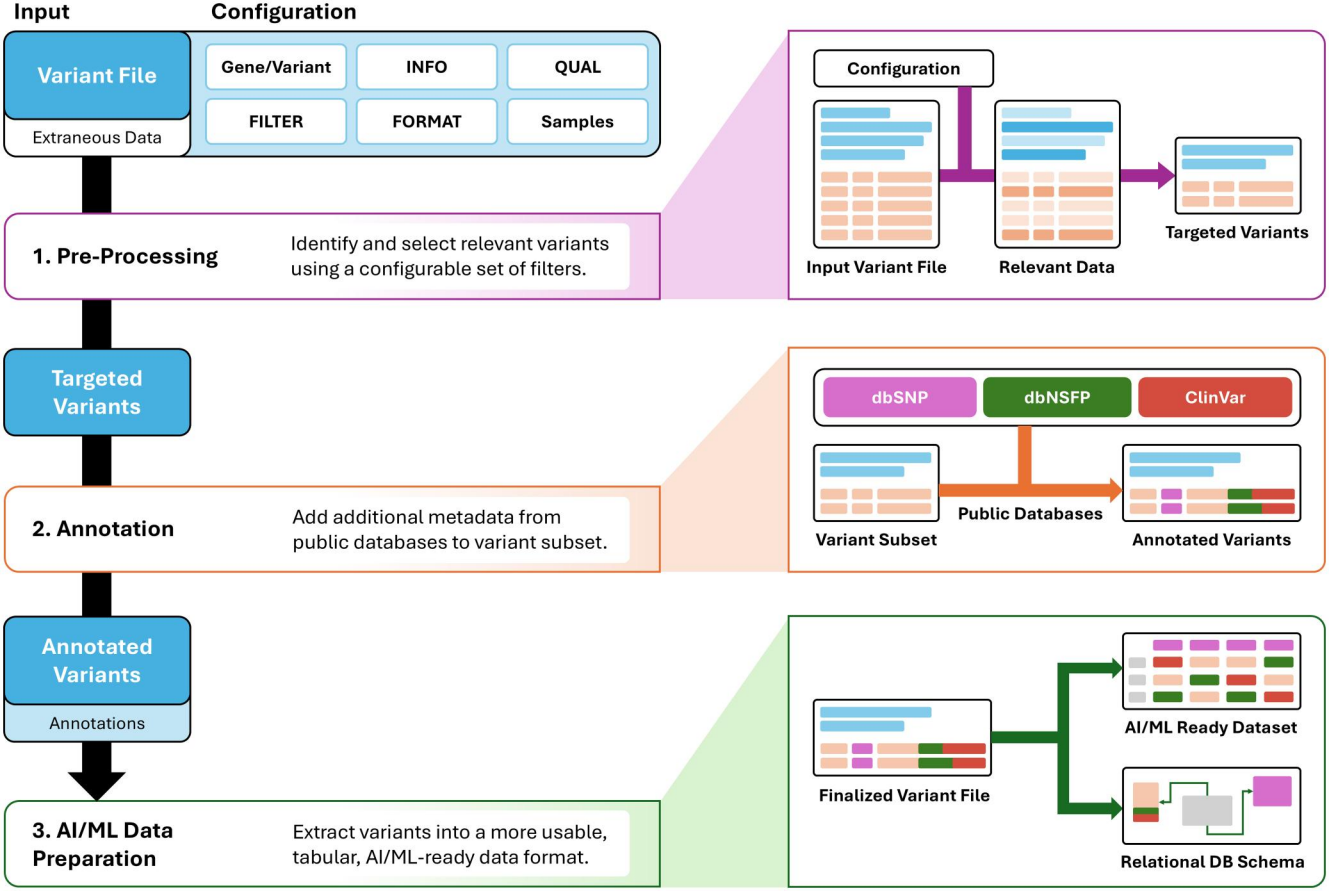
VAREANT pipeline and data transformation workflow. The overall workflow is divided into three steps, (i) Pre-Processing to support user in curating targeted datasets, (ii) Annotation to assist user in easily annotating variants files, and (iii) AI/ML Data Preparation offer feature for extracting VCF files into a more usable AI/ML ready data format.

VAREANT is split into three standalone modules: (i) Preprocessing, (ii) Variant Annotation, and (iii) AI/ML Data Preparation (Fig. 1). Each module may be used independently on custom datasets or chained together to robustly extract relevant variant data from a larger genomics dataset.

### 2.1 Preprocessing

To reduce overall complexity associated with genomic data management and analysis, it is important to successfully identify and minimize any extraneous data. To support the curation of these targeted genomic datasets, VAREANT implements a highly efficient and customizable filtering methodology for selecting maximally relevant variants and metadata. Through the application of different filters, the user has nuanced control over which data points are retained, including genes, quality scores, pathogenicity scores, sample genotype data, annotations, allele frequencies, etc. In maintaining compatibility with the VCF standard, VAREANT also properly handles missing values, such as quality scores or variant identifiers (IDs). Missing values in the VCF format is denoted by a dot (i.e. “.”). Notably, VAREANT enables the selective extraction of both variants by their rsIDs and genes by either gene symbols or Ensembl IDs (Martin *et al.* 2023). The latter is particularly useful when the target gene set is known *a priori*, such as in many clinical studies. In addition to extracting gene-related data, specific variant metadata important for subsequent analysis may also be retrieved using VAREANT. Large variant files with hundreds of thousands of variants inherently require large amounts of storage capacities. As such, VCF datasets are often heavily compressed in binary-encoded formats. VAREANT supports the user with both uncompressed and compressed formats (i.e. “.vcf” and “.vcf.gz,” respectively). As input, the user may provide any VCF dataset and VAREANT will automatically detect whether it is compressed and adjust its parsing strategies accordingly and efficiently. Supporting compressed datasets is crucial in addressing the large volume limitation of genomic datasets. Furthermore, heavily annotated variant files may contain hundreds of features per variant, amassing to large volumes of potentially unused data. Filtering these extraneous annotations can lead to drastic reduction of file sizes. Variant files may also contain information about multiple sequenced samples, such as read depth, read quality, or haplotype phasing. For targeted analyses or case/control studies where only a subset of this data is required for investigation, VAREANT can efficiently extract this information. All filtering criteria are defined through a single configuration file. For convenience, lists of gene symbols, Ensembl IDs, or variant ids may be provided directly or via an external file such as those generated by custom or third-party bioinformatic pipelines. VAREANT was developed to be performant in different environments with variable resources, by efficiently taking full advantage of the available computing hardware. Larger files are split into smaller manageable chunks and processed efficiently on multiple central processing units (CPUs) in parallel. Moreover, by dynamically streaming chunks into memory, VAREANT can maintain a minimal memory footprint. With this dynamic architecture, it is crucial to support the user in monitoring execution progress, troubleshooting errors, and replicating results. Hence, VAREANT creates detailed and timestamped logs on every execution, including any encountered errors or misconfigurations. We have open sourced our command line tool, written in Python, under a permissive and non-commercial GPLv3 license and made it publicly available on our GitHub. With its simple interface, it is accessible to researchers and clinicians lacking a computational background, requiring only a basic understanding of executing scripts. Details on installing and using VAREANT can be found in the Supplementary Material S1, VAREANT users guide.

### 2.2 Variant annotation

Variant annotation is the process of associating metadata from public databases with corresponding variant data. The choice of annotations can have a significant impact on the interpretation and conclusions of genomic analyses (McCarthy *et al.* 2014). Although many other annotation tools exist, such as VEP or ANNOVAR, VAREANT employs SnpEff and SnpSift due to its simplicity, portability, and efficiency (Cingolani 2022). For annotation databases, VAREANT currently supports dbSNP, dbNSFP, and ClinVar to identify clinical significance and functional pathogenicity scores for known variants. dbSNP is a public and centralized repository of genetic variation constituting primarily of single nucleotide polymorphisms (SNPs), the most common type of genetic variation (Sherry *et al.* 2001). Notably, dbSNP may be used to auto-generate IDs for every known variant in the dataset. This is particularly useful if the original VCF-formatted dataset has missing values in the ID column. However, dbSNP makes no distinction between neutral and pathogenic variants. For this purpose, dbNSFP can be used to provide 36 different functional pathogenicity scores aiding in determining variant deleteriousness (Liu *et al.* 2020). For example, SIFT and PolyPhen scores may be used to predict whether a mutation is likely to affect protein structure, or Combined Annotation-Dependent Depletion (CADD) (Rentzsch *et al.* 2019) and Eigen scores (Ionita-Laza *et al.* 2016) which integrate numerous functional annotations to generate a single ML-based deleterious metric. Effective pathogenicity scores are crucial for identifying variations of interest and discovering disease etiology. Lastly, ClinVar is a public archive of genetic variants and their significance in human disease (Landrum *et al.* 2018) and can be used as a basis for investigating variant-disease associations. Using VAREANT, the user may optionally annotate their datasets with any of these public databases depending on the study-specific needs. VAREANT supports the use of all versions of dbSNP, dbNSFP, and ClinVar, including the latest and most up to date releases. Using SnpEff and SnpSift, the user may also annotate using any other VCF-formatted annotation database of their choice. The necessary executables can be accessed from the bioconda repository, but VAREANT comes prepackaged with them for convenience. Since variant annotation is more time consuming for larger files, the user should filter their dataset using VAREANT before annotation to reduce the overall processing burden. While using VAREANT, at least one annotation database must be specified, or else variant annotation is entirely skipped. Moreover, to support and enable scientists with their own custom annotation pipelines, the user can integrate VAREANT’s standalone modules independently with their own tools and annotation databases, widening the scope of use cases for VAREANT. The modular design of VAREANT allows the user to augment and enhance their existing bioinformatic pipelines and support more complex and custom data preparation workflows.

### 2.3 AI/ML data preparation

AI/ML techniques show promise in progressing modern genomic analysis and personalized treatment by aiding scientists in understanding the genetic basis of disease (Ahmed *et al.* 2020, Ahmed 2022). However, extensive preprocessing is often required to prepare data in a format conducive to AI/ML analysis. Although the VCF format is effective at encoding variation data, it is not suitable for immediate use. To prepare variant files for subsequent AI/ML analysis, VAREANT supports the extraction and transformation of variant and sample data into a tabular AI/ML-ready structure. This tabular structure is preferred over other formats for AI/ML analysis. It enables the integration of clinical demographic information with genomic data and is well supported by various programming languages. With VAREANT, the user can extract sample-specific information into this AI/ML-ready format, including genotype quality, genotype probabilities, read depths, haplotype, etc. Employing AI/ML predictive techniques on these extracted datasets can offer a deeper understanding of how the samples are correlated. Additionally, for analyses where only the presence of a variant is important, the user can extract a simpler binary matrix representing whether each variant is present in a sample. In addition to a tabular format, we recognize that bioinformaticians often have their own data management pipeline. To better support these custom workflows, the user may also extract data into a JWES-compatible relational database (Ahmed *et al.* 2021), enabling them to integrate their datasets with existing structured query language (SQL) data management solutions. This relational structure is more convenient for organizing variant annotations with sample data. VAREANT was written in the Python programming language and requires at least Python 3.6 to be installed on the system. It is compatible with all major operating systems (OS) including Microsoft Windows, Apple MacOS, and Linux. For annotation, VAREANT uses SnpEff which requires Java to be installed on system. For AI/ML data preparation, the user must also install the Pandas and NumPy python packages. For handling compressed VCF datasets, VAREANT uses the pysam librarcy. We have open-sourced our tool to be accessible to the wider scientific community. Further installation and usage instructions can be found on our public GitHub as well as in the Supplementary Material S1, VAREANT users guide.

## 3 Results

### 3.1 Data collection

VAREANT has been successfully tested and validated in-house on varied datasets and environments. From a cohort of 96 patients, we carefully crafted 98 datasets. Primary analysis was performed on 96 of these datasets, which were sequenced from each of the individual patients. Computational validation was performed on the remaining two datasets which were separately curated as a combination of the other 96 datasets. Specifically, one of these curated datasets was smaller in size, but denser with CVD variants, while the other was much larger in size to computational validate VAREANT on large volumes of genomic data. The 96 samples were aggregated from two peer-reviewed studies investigating impactful genes and their associations with CVD, including atrial fibrillation (AF) and heart failure (HF). The first cohort contained 61 patients (Mhatre *et al.* 2023), and the second contained the remaining 35 (Venkat *et al.* 2023). Combined, 61 patients were male and 35 were female, aged between 24 and 94. Age and gender information for each patient is enumerated in Supplementary Material S2. VAREANT was used to preprocess and annotate each of these datasets ranging from 0.5 gigabytes (GB) to 52 GB in size. To further validate VAREANT, we derived a literature-based set of CVD-associated genes and variants to cross-reference the extracted results. We recently conducted a thorough review of literature published between 2009 and 2022 focused on integrative genomic approaches, common and rare genetic variant analyses for CVDs, and multi-ethnic studies (Patel *et al.* 2023). In that study, we identified a total of 214 variants from 190 genes associated with AF, and 28 variants from 26 genes associated with HF. This gene set of 216 genes was used as the primary criterion for filtering and annotation with VAREANT (Patel *et al.* 2023). Of these 216 genes, we identified and reported 136 genes (119 for AF and 17 for HF) present in our datasets, which are listed in Table 1. All filtered variants were manually reviewed to ensure consistency with the specified gene set. The original 96 samples consisted of 396 788 923 total variants, which was narrowed down by over a factor of 100 to 3 906 744 variants using VAREANT. Annotation on this targeted dataset was subsequently performed using dbSNP, dbNSFP, and ClinVar. dbSNP (dated 23 April 2018) was used to identify each variant by their rsIDs, and dbNSFP (v4.1a) and ClinVar (dated 08 July 2024) was used to determine pathogenicity and clinical significance of each variant.

**Table 1.** Gene-variants associated with the atrial fibrillation (AF) and heart failure (HF).

#	Gene	Variants (RS numbers)	Disease
1	AGBL4	rs11590635	AF
2	AKAP6	rs2145587; rs11156751	AF
3	ANXA4	rs3771537	AF
4	ARHGAP10	rs10213171	AF
5	ARHGAP26	rs6580277	AF
6	ASAH1	rs7508	AF
7	ATXN1	rs73366713	AF
8	BEST3	rs35349325	AF
9	C10orf11	rs11001667; rs10458660	AF
10	C10orf76	rs1044258	AF
11	C9orf3	rs4385527; rs10821415	AF
12	C9orf3(FBP1)	rs10821415	AF
13	C9orf3(FBP2)	rs10821415	AF
14	CAMK2D	rs55754224; rs6829664	AF
15	CAND2	rs6810325; rs4642101; rs7650482	AF
16	CASQ2	rs4484922; rs4073778	AF
17	CASZ1	rs880315; rs284277	AF
18	CAV1	rs11773845; rs3807989	AF
19	CDK6	rs11773884; rs56201652	AF
20	CDKN1A	rs3176326	AF
21	CEP68	rs2540949	AF
22	CFL2	rs73241997	AF
23	COG5	rs62483627	AF
24	CREB5	rs6462078; rs6462079	AF
25	CUL4A	rs35569628	AF
26	CUX2	rs6490029	AF
27	CYTH1	rs12604076	AF
28	DGKB	rs55734480	AF
29	DNAH10	rs12298484	AF
30	DPF3	rs74884082	AF
31	EPHA3	rs7632427; rs6771054	AF
32	ERBB4	rs35544454	AF
33	FAM13B	rs2967791	AF
34	FBN2	rs2012809	AF
35	FBRSL1	rs6560886	AF
36	FBXO32	rs62521286	AF
37	FRMD4B	rs17005647	AF
38	GMCL1	rs3771537	AF
39	GNB4	rs4855075; rs7612445	AF
40	GOPC	rs210632	AF
41	GOSR2	rs76774446	AF
42	GTF2I	rs74910854; rs35005436	AF
43	GYPC	rs28387148	AF
44	HAND2	rs10520260	AF
45	HCN4	rs7164883	AF
46	HIP1R	rs10773657	AF
47	IGF1R	rs12908437; rs4965430	AF
48	KCND3	rs12044963; rs1545300	AF
49	KCNH2	rs7789146	AF
50	KCNJ5	rs76097649; rs75190942	AF
51	KCNN2	rs716845; rs337711; rs337705	AF
52	KDM1B	rs34969716	AF
53	KIF3C	rs6546620; rs7578393	AF
54	KLHL3	rs2967791	AF
55	LHX3	rs2274115	AF
56	LINC00964	rs35006907	AF
57	LRIG1	rs2306272; rs34080181	AF
58	MAPT	rs242557	AF
59	MBD5	rs12992412	AF
60	MEX3C	rs8088085	AF
61	MIR30B	rs7460121	AF
62	MTSS1	rs35006907; rs35006907	AF
63	MYH7	rs28631169; rs422068	AF
64	MYO18B	rs133902	AF
65	MYOCD	rs72811294	AF
66	MYOZ1	rs10824026	AF
67	NACA	rs7978685; rs2860482	AF
68	NAV2	rs1822273; rs10741807	AF
69	NKX2-5	rs6882776	AF
70	NME5	rs2040862	AF
71	NR3C1	rs6580277	AF
72	NUCKS1	rs4951261; rs4951258	AF
73	OPN1SW	rs55985730	AF
74	PAK2	rs9872035	AF
75	PCM1	rs7508	AF
76	PHLDB2	rs17490701; rs10804493	AF
77	PKP2	rs12809354	AF
78	PLN	rs4946333; rs89107	AF
79	POLR2A	rs9899183	AF
80	PPFIA4	rs10753933; rs17461925	AF
81	PPP2R3A	rs1278493	AF
82	PSMB7	rs10760361	AF
83	PTK2	rs6993266; rs6994744	AF
84	RBM20	rs10749053	AF
85	REEP3	rs7919685; rs12245149	AF
86	RPS2	rs2286466	AF
87	SCMH1	rs2885697	AF
88	SCN10A	rs6790396; rs6800541	AF
89	SH3PXD2A	rs2047036	AF
90	SIRT1	rs7096385	AF
91	SLC27A6	rs2012809	AF
92	SLC35F1	rs17079881; rs4946333; rs89107; rs3951016	AF
93	SLC9B1	rs3960788; rs10006327	AF
94	SLIT3	rs12188351	AF
95	SMAD7	rs9953366	AF
96	SNRNP27	rs10165883; rs6747542	AF
97	SNX6	rs73241997	AF
98	SPATS2L	rs295114; rs3820888	AF
99	SSPN	rs113819537; rs17380837	AF
100	SUN1	rs11768850	AF
101	SYNE2	rs2738413; rs1152591	AF
102	SYNPO2L	rs60212594; rs10824026	AF
103	TBX5	rs883079; rs10507248	AF
104	TEX41	rs67969609	AF
105	THRB	rs73032363; rs73041705	AF
106	TNFSF12	rs9899183	AF
107	TTN	rs35504893; rs2288327	AF
108	TTN-AS1	rs2288327	AF
109	TUBA8	rs465276; rs464901	AF
110	USP3	rs62011291	AF
111	UST	rs117984853	AF
112	WDR1	rs3822259	AF
113	WNT8A	rs2967791; rs2040862	AF
114	XPO1	rs6742276	AF
115	XPO7	rs7846485; rs7834729	AF
116	XXYLT1	rs60902112	AF
117	ZFHX3	rs2359171; rs2106261	AF
118	ZNF462	rs4743034	AF
119	ZPBP2	rs11658278	AF
120	AGAP5	rs4746140	HF
121	ATXN2	rs4766578	HF
122	BAG3	rs17617337; rs2234962	HF
123	CDKN1A	rs4135240	HF
124	CDKN2B-AS1	rs1556516	HF
125	CELSR2	rs660240	HF
126	KLHL3	rs11745324	HF
127	LINC00964	rs35006907	HF
128	LPA	rs55730499; rs140570886	HF
129	MAP7D1	rs272825; rs272832	HF
130	MTSS1	rs35006907; rs34866937; rs35006907	HF
131	NMB	rs2175567; rs17598603	HF
132	SCN5A	rs1805126	HF
133	SH2B3	rs7310615	HF
134	SURF1	rs600038	HF
135	SYNPOL2L	rs4746140	HF
136	TTN	rs2042995; rs2255167	HF

This table includes genes, variant, and disease information.

### 3.2 Case study #1: heart failure

From our processed dataset, we first identified the total number of variants on genes associated with HF. Specifically, 220 348 variants of the 3.9 million variants filtered by VAREANT belonged to HF-associated genes, according to our filtering criterion. We then explored the individual annotations to identify variants with known clinical significance and pathogenic effects. 12 373 of these variants reported clinical significance and pathogenicity annotations, most of which were marked benign or with uncertain significance. The remaining novel variants lacked any annotations. In total, nine unique variants were successfully identified from ClinVar annotations as having some pathogenic association in a GWAS study or being a risk factor for disease. Specifically, deleterious variants were labelled as either “pathogenic,” “likely pathogenic,” “risk factor,” or “association,” as described by ClinVar’s variant classification guidelines (Landrum *et al.* 2018). Specifically, rs1063192 was indicated to be likely pathogenic; rs977371848, rs12740374, rs947073006 were marked as having some association to GWAS a study; and rs1333049, rs10757274, rs1421085, rs4977574 were identified as being risk factors. From our original set of 28 HF-associated variants, 21 were present in our dataset and all were marked benign/likely benign.

Significance of each variant in HF and CVDs in general was reviewed through authentic literature. We discovered that rs1063192 has been studied to have a positive association with myocardial infarction (MI) in Han Chinese male patients (Yang *et al.* 2009); rs1421085 has associations with childhood and adult obesity (Dina *et al.* 2007, Price *et al.* 2008); rs1333049 has been studied to have associations with coronary artery disease (CAD) in Caucasian (Wellcome Trust Case Control Consortium 2007), Japanese (Hinohara *et al.* 2008), and Korean populations (Hinohara *et al.* 2008), and associations with MI in German populations (Samani *et al.* 2007); rs4977574 shows change in African and Middle-Eastern populations for type 2 diabetes and CAD (Silander *et al.* 2009); rs10757274 has been previously associated with MI in Italian (Shen *et al.* 2008a), as well as CAD in Korean populations (Shen *et al.* 2008b); rs12740374 was studied to be highly associated with low-density lipoprotein cholesterol (Musunuru *et al.* 2010). The remaining variants were not previously found to have associations with HF. An aggregate of these variants, their prevalence in our dataset, and their clinical significance is enumerated in Table 2.

**Table 2.** Disease-associated variants identified by the ClinVar.

Gene	Variant (RS number)	Frequency (in 96 sample cohort)	Clinical significance based on ClinVar	Disease names based on ClinVar
COG5	rs1449966934	1	Pathogenic	COG5 congenital disorder of glycosylation
CDKN2B-AS1	rs1063192	76	Likely pathogenic	Malignant tumor of breast, three vessel coronary disease
	rs1333049	70	Risk factor	Three vessel coronary disease
	rs4977574	70	Risk factor	Three vessel coronary disease
	rs10757274	71	Risk factor	Three vessel coronary disease
FTO	rs1421085	70	Risk factor	Obesity (BMIQ14) susceptibility
ABO	rs947073006	90	Association	ABO blood group system
	rs977371848	90	Association	ABO blood group system
	rs992108547	90	Association	ABO blood group system
ARNT2	rs3901896	55	Association	Pulmonary disease susceptibility
	rs8041826	31	Association	Pulmonary disease susceptibility
CDK6	rs42034	40	Association	Bechet disease
	rs2282983	65	Association	Bechet disease
CELSR2	rs12740374	32	Association	LDL cholesterol
CREB5	rs4722804	30	Association	Vascular endothelial growth factor inhibitor response
SLC35F1	rs11153718	34	Association	Vascular endothelial growth factor inhibitor response

This table enumerates genes, RS Number, frequency in cohort, pathogenic scoring based on ClinVar, and associated diseases for 16 variants. These variants were identified by ClinVar as having some known association to diseases.

### 3.3 Case study #2: atrial fibrillation

Of the over 3.9 million variants filtered using VAREANT, the remaining 3 686 396 belonged to genes associated with AF. 101 745 of these variants were annotated to have some clinical significance, and 7 unique variants indicating some pathogenic clinical significance (Landrum *et al.* 2018). Namely, rs1449966934 was marked pathogenic, and rs8041826, rs4722804, rs2282983, rs11153718, rs3901896, rs42034 were all identified as having some previously studied GWAS association. From our original set of 214 AF-associated variants, 151 variants were present in our dataset, all of which were also annotated as benign/likely benign. These variants are enumerated in Table 2. To explore the relationship of the pathogenic variants with CVDs, we reviewed authentic literature for each variant. Notably, rs42034 was studied to have negative associations with Bechet’s disease (an inflammatory disease) in the Han Chinese population (Cai *et al.* 2022). The other six variants were not previously explored in literature, but rs1449966934 was annotated to have associations with the congenital disorder glycosylation. Further study is required to identify any potential functional associations these variants may have with CVDs.

### 3.4 Computational validation

Performance was extensively tested and benchmarked on different OS (MacOS, Linux, Windows), on different hardware configurations (4 CPUs and 8 GB memory, 12 CPUs, and 32 GB memory), in high performance computing (HPC) and local desktop environments, and in single-processor and multi-processor modes. The results of each configuration for the two curated datasets (i.e. the variant-dense dataset and the large volume dataset) as well as one of the 96 patient-specific datasets are listed in Supplementary Material S2. The variant-dense dataset was 0.5 GB in size, while the large volume dataset was 52 GB. The patient-specific dataset selected for computational validation was 2.6 GB large. This range of file sizes was carefully selected to validate VAREANT’s efficacy on variable-sized datasets. We have tested and reproduced results on all major OS including Microsoft Windows, Apple MacOS, and Linux. In Supplementary Material S2, we have aggregated and reported results from execution on a HPC environment running Linux CentOS version 7.9.2009. Notably, VAREANT successfully filtered out over 97% of the combined dataset (0.5 GB) and over 99% of both the 2.6 GB and 52 GB datasets. VAREANT also performs better on more powerful hardware, completing filtering nearly twice as fast on the single 2.6 GB patient-specific dataset when using 12 CPUs over 4 CPUs. The large 52 GB dataset, with nearly 83 million variants, was successfully filtered within 2 minutes when using all available CPU cores. Moreover, on a single processor, limited memory does not appear to pose a noticeable bottleneck. The performance metrics also effectively delineate the impact that filtering with VAREANT has on subsequent annotation. Annotating the medium-sized 2.6 GB dataset using dbSNP, dbNSFP, and ClinVar took over 3 hours on an M2 MacBook, which was reduced to merely 16 seconds after first filtering the dataset for relevant variants only. The smaller yet variant-dense dataset also experienced significant improvements in annotation from nearly 15 minutes before filtering to only 44 seconds after filtering. Annotation on the large 52 GB dataset was only performed after preprocessing due to limited resource availability, taking over 54 minutes to complete. By extrapolating results, we can estimate that annotating the original unfiltered dataset could have taken nearly 4 weeks. Although these results are subject to the hardware resources and content of the dataset, we recommend running VAREANT in its multi-processor mode to take full advantage of provided hardware, and to filter before annotation to minimize computational times. This configuration is the default.

## 4 Discussion

The curation of high-quality, relevant genomic datasets is necessary to facilitate genomic analysis that can transform our current understanding of gene-disease relationships and improve our ability to provide personalized treatment options for patients. Moreover, it may help minimize difficulties associated with genomic data management, such as the high storage capacities needed to handle large volumes of data (Stephens *et al.* 2015) and the time-consuming processing (Alvarez *et al.* 2021). In this study, we aim to address some of these challenges by presenting VAREANT, a highly configurable and accessible bioinformatics tool to process large volumes of variant data into targeted datasets suitable for subsequent analysis. Here, we demonstrated the efficacy of VAREANT in a case study of variant data of CVD patients. We curated a gene set validated from literature, we successfully identified targeted variants with associations with CVD diseases, such as CAD and MI.

VAREANT was developed alongside a recent study we conducted wherein we applied AI/ML techniques to predict CVD in a patient population based on integrated RNA-Seq expression data and genomics variant data (DeGroat *et al.* 2024). The study, challenged us in the processing and preparation of hundreds of GBs of genomics data in a format suitable for AI/ML analysis, prompting the need to curate a targeted, AI/ML-ready (Ahmed *et al.* 2024), CVD dataset. Although VAREANT was originally developed to support this specific study, we recognized the broader potential impact of such bioinformatic applications and developed it into a publicly available tool for the wider scientific community. Recently, we also developed and proposed IntelliGenes, a novel AI/ML framework for disease prediction and biomarker discovery in patients using multi-omics data (DeGroat *et al.* 2023, Narayanan *et al.* 2024). VAREANT is well suited to support the user with complex, multi-modal AI/ML analysis using tools like IntelliGenes. By chaining JWES, VAREANT, and IntelliGenes, the user can streamline the full genomic data transformation workflow. VAREANT may also be expanded to include more fine-grained filtering options for better data extraction. More advanced features, such as the ability to extract variants in the flanking and regulatory regions of a gene, may be implemented in the future to allow for the curation of even more targeted datasets for highly focused analyses. Here, we demonstrate that accessible bioinformatic tools such as VAREANT, aid in preparing large volumes of data, and are critical and effective in predictive genomic analyses.

## Supplementary Material

vbae210_Supplementary_Data

## Data Availability

The open-source code of VAREANT is available on GitHub < https://github.com/drzeeshanahmed/Gene_VAREANT >.

## References

[vbae210-B1] Ahmed Z , Mohamed K, Zeeshan S et al Artificial intelligence with multi-functional machine learning platform development for better healthcare and precision medicine. Database (Oxford) 2020;2020:baaa010. 10.1093/database/baaa010 32185396 PMC7078068

[vbae210-B2] Ahmed Z , Renart EG, Mishra D et al JWES: a new pipeline for whole genome/exome sequence data processing, management, and gene-variant discovery, annotation, prediction, and genotyping. FEBS Open Bio 2021;11:2441–52. 10.1002/2211-5463.13261 PMC8409305 34370400

[vbae210-B3] Ahmed Z. Precision medicine with multi-omics strategies, deep phenotyping, and predictive analysis. Prog Mol Biol Transl Sci 2022;190:101–25. 10.1016/bs.pmbts.2022.02.002 36007996

[vbae210-B4] Ahmed Z , Wan S, Zhang F et al Artificial intelligence for omics data analysis. BMC Methods 2024;1. 10.1186/s44330-024-00004-5

[vbae210-B5] Alvarez RV , Mariño-Ramírez L, Landsman D. Transcriptome annotation in the cloud: complexity, best practices, and cost. Gigascience 2021;10:giaa163. 10.1093/gigascience/giaa163 33511996 PMC7845158

[vbae210-B6] Cai S , Zhang J, Zhou C et al Association of CDK6 gene polymorphisms with Behcet’s disease in a Han Chinese population. Exp Eye Res 2022;223:109203. 10.1016/j.exer.2022.109203 35921963

[vbae210-B7] Cingolani P. Variant annotation and functional prediction: SnpEff. Methods Mol Biol 2022;2493:289–314. 10.1007/978-1-0716-2293-3_19 35751823

[vbae210-B8] Danecek P , Auton A, Abecasis G, et al; 1000 Genomes Project Analysis Group. The variant call format and VCFtools. Bioinformatics 2011;27:2156–8. 10.1093/bioinformatics/btr330 21653522 PMC3137218

[vbae210-B9] DeGroat W , Abdelhalim H, Peker E et al Multimodal AI/ML for discovering novel biomarkers and predicting disease using multi-omics profiles of patients with cardiovascular diseases. Sci Rep 2024;14:26503. Published 3 November 2024. 10.1038/s41598-024-78553-6 39489837 PMC11532369

[vbae210-B10] DeGroat W , Mendhe D, Bhusari A et al IntelliGenes: a novel machine learning pipeline for biomarker discovery and predictive analysis using multi-genomic profiles. Bioinformatics 2023;39:btad755. 10.1093/bioinformatics/btad755 38096588 PMC10739559

[vbae210-B11] Dina C , Meyre D, Gallina S et al Variation in FTO contributes to childhood obesity and severe adult obesity. Nat Genet 2007;39:724–6. 10.1038/ng2048 17496892

[vbae210-B12] Hinohara K , Nakajima T, Takahashi M et al Replication of the association between a chromosome 9p21 polymorphism and coronary artery disease in Japanese and Korean populations. J Hum Genet 2008;53:357–9. 10.1007/s10038-008-0248-4 18264662

[vbae210-B13] Ionita-Laza I , McCallum K, Xu B et al A spectral approach integrating functional genomic annotations for coding and noncoding variants. Nat Genet 2016;48:214–20. 10.1038/ng.3477 26727659 PMC4731313

[vbae210-B14] Landrum MJ , Lee JM, Benson M et al ClinVar: improving access to variant interpretations and supporting evidence. Nucleic Acids Res 2018;46:D1062–D1067. 10.1093/nar/gkx1153 29165669 PMC5753237

[vbae210-B15] Lin Q , Tam PK, Tang CS. Artificial intelligence-based approaches for the detection and prioritization of genomic mutations in congenital surgical diseases. Front Pediatr 2023;11:1203289. Published 1 August 2023. 10.3389/fped.2023.1203289 37593442 PMC10429173

[vbae210-B16] Liu X , Li C, Mou C et al dbNSFP v4: a comprehensive database of transcript-specific functional predictions and annotations for human nonsynonymous and splice-site SNVs. Genome Med 2020;12:103. Published 2 December 2020. 10.1186/s13073-020-00803-9 33261662 PMC7709417

[vbae210-B17] Musunuru K , Strong A, Frank-Kamenetsky M et al From noncoding variant to phenotype via SORT1 at the 1p13 cholesterol locus. Nature 2010;466:714–9. 10.1038/nature09266 20686566 PMC3062476

[vbae210-B18] McKenna A , Hanna M, Banks E et al The genome analysis toolkit: a MapReduce framework for analyzing next-generation DNA sequencing data. Genome Res 2010;20:1297–303. 10.1101/gr.107524.110 20644199 PMC2928508

[vbae210-B19] McLaren W , Gil L, Hunt SE et al The ensembl variant effect predictor. Genome Biol 2016;17:122. Published 6 June 2016. 10.1186/s13059-016-0974-4 27268795 PMC4893825

[vbae210-B20] Martin FJ , Amode MR, Aneja A et al Ensembl 2023. Nucleic Acids Res 2023;51:D933–D941. 10.1093/nar/gkac958 36318249 PMC9825606

[vbae210-B21] McCarthy DJ , Humburg P, Kanapin A et al Choice of transcripts and software has a large effect on variant annotation. Genome Med 2014;6:26. Published 31 March 2014. 10.1186/gm543 24944579 PMC4062061

[vbae210-B22] Mhatre I , Abdelhalim H, Degroat W et al Functional mutation, splice, distribution, and divergence analysis of impactful genes associated with heart failure and other cardiovascular diseases. Sci Rep 2023;13:16769. Published 5 October 2023. 10.1038/s41598-023-44127-1 37798313 PMC10556087

[vbae210-B23] Narayanan R , DeGroat W, Mendhe D et al *IntelliGenes*: interactive and user-friendly multimodal AI/ML application for biomarker discovery and predictive medicine. Biol Methods Protoc 2024;9:bpae040. Published 29 May 2024. 10.1093/biomethods/bpae040 38884000 PMC11176709

[vbae210-B24] Patel KK , Venkatesan C, Abdelhalim H et al Genomic approaches to identify and investigate genes associated with atrial fibrillation and heart failure susceptibility. Hum Genomics 2023;17:47. Published 3 June 2023. 10.1186/s40246-023-00498-0 37270590 PMC10239148

[vbae210-B25] Price RA , Li WD, Zhao H. FTO gene SNPs associated with extreme obesity in cases, controls and extremely discordant sister pairs. BMC Med Genet 2008;9:4. Published 24 January 2008. 10.1186/1471-2350-9-4 18218107 PMC2254593

[vbae210-B26] Rentzsch P , Witten D, Cooper GM et al CADD: predicting the deleteriousness of variants throughout the human genome. Nucleic Acids Res 2019;47:D886–D894. 10.1093/nar/gky1016 30371827 PMC6323892

[vbae210-B27] Samani NJ , Erdmann J, Hall AS, et al; WTCCC and the Cardiogenics Consortium. Genomewide association analysis of coronary artery disease. N Engl J Med 2007;357:443–53. 10.1056/NEJMoa072366 17634449 PMC2719290

[vbae210-B28] Schadt EE , Linderman MD, Sorenson J et al Computational solutions to large-scale data management and analysis. Nat Rev Genet 2010;11:647–57. 10.1038/nrg2857 20717155 PMC3124937

[vbae210-B29] Sherry ST , Ward MH, Kholodov M et al dbSNP: the NCBI database of genetic variation. Nucleic Acids Res 2001;29:308–11. 10.1093/nar/29.1.308 11125122 PMC29783

[vbae210-B30] Shen GQ , Rao S, Martinelli N et al Association between four SNPs on chromosome 9p21 and myocardial infarction is replicated in an Italian population. J Hum Genet 2008a;53:144–50. 10.1007/s10038-007-0230-6 18066490

[vbae210-B31] Shen GQ , Li L, Rao S et al Four SNPs on chromosome 9p21 in a South Korean population implicate a genetic locus that confers high cross-race risk for development of coronary artery disease. Arterioscler Thromb Vasc Biol 2008b;28:360–5. 10.1161/ATVBAHA.107.157248 18048766

[vbae210-B32] Silander K , Tang H, Myles S et al Worldwide patterns of haplotype diversity at 9p21.3, a locus associated with type 2 diabetes and coronary heart disease. Genome Med 2009;1:51. Published 12 May 2009. 10.1186/gm51 19463184 PMC2689443

[vbae210-B33] Stephens ZD , Lee SY, Faghri F et al Big data: astronomical or genomical? PLoS Biol 2015;13:e1002195. Published 7 July 2015. 10.1371/journal.pbio.1002195 26151137 PMC4494865

[vbae210-B34] Uffelmann E , Huang QQ, Munung NS et al Genome-wide association studies. Nat News 2021. 10.1038/s43586-021-00056-9

[vbae210-B35] Venkat V , Abdelhalim H, DeGroat W et al Investigating genes associated with heart failure, atrial fibrillation, and other cardiovascular diseases, and predicting disease using machine learning techniques for translational research and precision medicine. Genomics 2023;115:110584. 10.1016/j.ygeno.2023.110584 36813091

[vbae210-B36] Wellcome Trust Case Control Consortium Genome-wide association study of 14,000 cases of seven common diseases and 3,000 shared controls. Nature 2007;447:661–78. 10.1038/nature05911 17554300 PMC2719288

[vbae210-B37] Yang XC , Zhang Q, Chen ML et al MTAP and CDKN2B genes are associated with myocardial infarction in Chinese Hans. Clin Biochem 2009;42:1071–5. 10.1016/j.clinbiochem.2009.02.021 19272367

