# Supplementary material for: VAREANT: a bioinformatics application for gene variant reduction and annotation
Source: Bioinform Adv. 2024 Dec 31;5(1):vbae210. doi: 10.1093/bioadv/vbae210 (PMC11802749; doi:10.1093/bioadv/vbae210)
Supplement: vbae210_Supplementary_Data [file vbae210_supplementary_data.zip › Supplementary_2_Data_and_Performance.pdf]

Supplementary Material# 2

VAREANT: Patient Demographic Data and Performance Metrics

Authors

Rishabh Narayanan[1], William DeGroat[1], Elizabeth Peker[1], Saman Zeeshan[2], and Zeeshan Ahmed[1, 3, *]

Affiliations

1. Rutgers Institute for Health, Health Care Policy and Aging Research, Rutgers, The State University of New Jersey, 112 Paterson St, New Brunswick, 08901, NJ, USA.

2. Department of Biomedical and Health Informatics, UMKC School of Medicine, 2411 Holmes Street, Kansas City, 64108, MO, USA.

3. Department of Medicine, Division of Cardiovascular Diseases and Hypertension, Robert Wood Johnson Medical School, Rutgers Health, 125 Paterson St, New Brunswick, NJ, 08901, USA.

***Corresponding author:** Zeeshan Ahmed, Rutgers Institute for Health, Health Care Policy and Aging Research, Rutgers University, 112 Paterson Street, New Brunswick, 08901, NJ, USA. (zahmed@ifh.rutgers.edu).

Patient Demographics

The case studies outlined in the manuscript were performed on a curated cohort of 96 samples, aggregated from 2 separate cardiovascular disease (CVD) studies. The following two tables (S. Table 1A, S. Table 1B) outline the demographic information (e.g. gender, age) for each patient.

#	ID	Gender	Age
1	1065	Female	51
2	1071	Female	52
3	1064	Female	54
4	1114	Female	54
5	1097	Female	57
6	1108	Female	57
7	1075	Female	59
8	1082	Female	59
9	1116	Female	63
10	1069	Female	65
11	1088	Female	65
12	1084	Female	69
13	1087	Female	69
14	1093	Female	70
15	1105	Female	71
16	1058	Female	72
17	1078	Female	72
18	1074	Female	81
19	1111	Female	86
20	1073	Female	89
21	1072	Female	91

22	1076	Male	45
23	1089	Male	55
24	1070	Male	57
25	1081	Male	57
26	1060	Male	58
27	1096	Male	59
28	1113	Male	60
29	1067	Male	62
30	1092	Male	62
31	1085	Male	64
32	1094	Male	64
33	1101	Male	64
34	1086	Male	65
35	1063	Male	66
36	1095	Male	66
37	1117	Male	66
38	1062	Male	67
39	1099	Male	67
40	1115	Male	67
41	1061	Male	70
42	1068	Male	70
43	1090	Male	70
44	1102	Male	71
45	1112	Male	72
46	1077	Male	73

47	1104	Male	73
48	1109	Male	75
49	1091	Male	77
50	1059	Male	79
51	1106	Male	79
52	1103	Male	80
53	1110	Male	80
54	1100	Male	81
55	1066	Male	82
56	1098	Male	83
57	1107	Male	84
58	1083	Male	85
59	1080	Male	86
60	1118	Male	88
61	1079	Male	92

S. Table 1A. Patient Demographics (Cohort A). This table enumerates sample ID, gender, and age of 61 patients [1], sorted by gender then by age. There are 21 females (colored orange), and 40 males (colored blue).

#	ID	Gender	Age
1	BR2-1228	Female	56
2	BR2-1154	Female	58
3	BR2-1421	Female	58
4	BR2-1094	Female	61
5	BR2-1267	Female	69
6	BR2-922	Female	72
7	BR2-738	Female	73
8	BR2-765	Female	74
9	BR2-1572	Female	76
10	BR2-821	Female	80
11	BR2-747	Female	83
12	BR2-860	Female	84
13	BR2-986	Female	89
14	BR2-731	Female	90
15	BR2-1343	Male	29
16	BR2-1506	Male	51
17	BR2-975	Male	58
18	BR2-1007	Male	58
19	BR2-781	Male	62
20	BR2-810	Male	62
21	BR2-1294	Male	62
22	BR2-833	Male	67
23	BR2-1218	Male	68
24	BR2-1366	Male	69

25	BR2-1282	Male	70
26	BR2-1563	Male	71
27	BR2-890	Male	72
28	BR2-995	Male	72
29	BR2-1381	Male	76
30	BR2-774	Male	77
31	BR2-745	Male	78
32	BR2-1273	Male	86
33	BR2-1090	Male	94
34	BR2-1095	Male	96
35	BR2-1606	Male	63

S. Table 1B. Patient Demographics (Cohort B). This table enumerates sample ID, gender, and age of 34 patients [2], sorted by gender then by age. There are 14 females (colored orange), and 20 males (colored blue).

VAREANT Performance Metrics

The following three tables (S. Table 2A, S. Table 2B, S. Table 2C) detail numerous statistics about the performance of each of the three modules of *VAREANT* on our custom datasets in a high-performance computing environment running Linux CentOS version 7.9.2009.

File Size (Before)	527 MB	2.6 GB	52 GB
File Size (After)	15 MB	1.2 MB	806 MB
# of Variants (Before)	99,777	4,051,911	82,996,009
# of Variants (After)	2,931	798	746,082
Duration (4 CPUs + 8 GB, Single-threaded)	6.9 s	96 s	N/A
Duration (4 CPUs + 8 GB, Multi-threaded)	4.3 s	28.1 s	N/A
Duration (12 CPUs + 32 GB, Single-threaded)	6.8 s	95 s	12 m 26 s
Duration (12 CPUs + 32 GB, Multi-threaded)	3.9 s	14.1 s	2 m 8 s

S. Table 2A. Performance of *VAREANT* Pre-Processing. This table outlines performance metrics of *VAREANT's* Pre-Processing module on three variable sized datasets (527 MB, 2.6 GB, 52 GB). It details statistics about the dataset before filtering, as compared to after filtering. It also lists the average processing duration in 4 different hardware environments (results subject to hardware and dataset itself). Due to the resource requirements, preprocessing was only performed on the largest dataset in the most performant configuration (12 CPUs + 32 GB memory).

File Size	527 MB	2.6 GB	52 GB
Duration (Without filtering)	14 m 45 s	3 h 37 m 11 s	N/A
Duration (With filtering)	44 s	16 s	54 m 49 s
File Size (Without filtering)	579 MB	3.7 GB	N/A
File Size (With filtering)	16 MB	1.4 MB	961 MB

S. Table 2B. Performance of *VAREANT* Annotation. This table outlines performance metrics of *VAREANT's* Annotation module on three variable sized datasets (527 MB, 2.6 GB, 52 GB). It details statistics about the size of the datasets and processing duration on the original unfiltered dataset, as compared to after filtering with *VAREANT*. Due to the large resource requirements, annotation was only performed after filtering. Extrapolating results provides a duration estimate of 4 weeks on the unfiltered dataset.

File Size	527 MB	2.6 GB	52 GB
Duration (Without filtering)	1.2 s	0.74 s	N/A
Duration (With filtering)	0.9 s	0.7 s	1 m 24 s
Tabular File Size (Without filtering)	20 MB	11 KB	N/A
Tabular File Size (With filtering)	569 KB	3.2 KB	8.9 MB
Relational DB Size (Without filtering)	2.3 GB	11.2 MB	N/A
Relational DB Size (With filtering)	36 MB	3.8 MB	9.4 GB

S. Table 2C. Performance of *VAREANT* AI/ML Data Preparation. This table outlines performance metrics of *VAREANT's* AI/ML Data Preparation module on three variable sized datasets (527 MB, 2.6 GB, 52 GB). It enumerates the sizes of the extracted AI/ML ready datasets, as well as the general processing duration. Due to the large resource requirements, extraction was only performed after filtering.

References

1. Venkat, V., Abdelhalim, H., DeGroat, W., Zeeshan, S., & Ahmed, Z. (2023). Investigating genes associated with heart failure, atrial fibrillation, and other cardiovascular diseases, and predicting disease using machine learning techniques for translational research and precision medicine. Genomics, 115(2), 110584. https://doi.org/10.1016/j.ygeno.2023.110584
2. Mhatre, I., Abdelhalim, H., Degroat, W., Ashok, S., Liang, B. T., & Ahmed, Z. (2023). Functional mutation, splice, distribution, and divergence analysis of impactful genes associated with heart failure and other cardiovascular diseases. Scientific reports, 13(1), 16769. https://doi.org/10.1038/s41598-023-44127-1
